# Early insights into the genome sequence of *Uromyces fabae*

**DOI:** 10.3389/fpls.2014.00587

**Published:** 2014-10-29

**Authors:** Tobias Link, Christian Seibel, Ralf T. Voegele

**Affiliations:** Fachgebiet Phytopathologie, Institut für Phytomedizin, Fakultät Agrarwissenschaften, Universität HohenheimStuttgart, Germany

**Keywords:** *Uromyces fabae*, rust fungus, genome survey, genome size, candidate effectors, RTP1 homologs

## Abstract

*Uromyces fabae* is a major pathogen of broad bean, *Vicia faba*. *U. fabae* has served as a model among rust fungi to elucidate the development of infection structures, expression and secretion of cell wall degrading enzymes and gene expression. Using *U. fabae*, enormous progress was made regarding nutrient uptake and metabolism and in the search for secreted proteins and effectors. Here, we present results from a genome survey of *U. fabae*. Paired end Illumina sequencing provided 53 Gb of data. An assembly gave 59,735 scaffolds with a total length of 216 Mb. K-mer analysis estimated the genome size to be 329 Mb. Of a representative set of 23,153 predicted proteins we could annotate 10,209, and predict 599 secreted proteins. Clustering of the protein set indicates families of highly likely effectors. We also found new homologs of RTP1p, a prototype rust effector. The *U. fabae* genome will be an important resource for comparative analyses with *U. appendiculatus* and *P. pachyrhizi* and provide information regarding the phylogenetic relationship of the genus *Uromyces* with respect to other rust fungi already sequenced, namely *Puccinia graminis* f. sp. *tritici, P. striiformis* f. sp. *tritici, Melampsora lini*, and *Melampsora larici-populina*.

## Introduction

The order Pucciniales is dominated by two genera: *Puccinia* with about 4,000 species and *Uromyces* with around 600 species (Maier et al., 2003). Though the phylogeny of the two genera has not been entirely disentangled, there is a clear tendency that *Puccinia* species mainly infect grass hosts, whereas *Uromyces* species seem to be concentrated on legumes. There is now sequence information available for two species within the genus *Puccinia* [*Puccinia graminis* f. sp. *tritici* (*Pgt*), and *Puccinia striiformis* f. sp. *tritici* (*Pst*)] (Duplessis et al., 2011; Cantu et al., 2013; Zheng et al., 2013). Recently, re-sequencing of several *Pst* strains was undertaken, enabling the search for accelerated evolution among secreted proteins, an interesting means for identifying highly likely effector candidates (Cantu et al., 2013). At the same time no genome information is available for the second largest genus *Uromyces*. Therefore, a reference genome for *Uromyces* enabling comparisons between these two closely related genera would be highly desirable.

Rust fungi cannot truly be called model species since their obligate biotrophic lifestyle makes basic research on these species very difficult. General information regarding fungi or basidiomycetes can much easier be obtained with other species. Among the 7,000 rust fungi, research is concentrated on a very limited selection of species. One reason that brought these species into focus is the high economic losses associated with them. This is true for the cereal rusts *Pgt* and *Pst*, or the Asian Soybean Rust, *Phakopsora pachyrhizi*. The other reason why some rust species are prominent in research is their historical significance. The most important example here is *Melampsora lini* on *Linum usitatissimum*, the system upon which Flor developed the gene-for-gene hypothesis (Flor, 1956). These early successes have been picked up in modern molecular research, for example making the connection between Avr genes and proteins secreted from haustoria (Catanzariti et al., 2006), or providing information on the interaction between Avr and R-proteins (Dodds et al., 2006), up to contributions as to how effectors may reach their targets (Rafiqi et al., 2010).

Among *Uromyces* species a similar role can be assigned to *U. fabae*. Over the years especially morphologic studies using light and electron microscopy contributed to the elucidation of infection structures of rust fungi (Kapooria and Mendgen, 1985). Later, biochemical studies contributed to the understanding of the physiology of early infection (Deising et al., 1991). The development of a method to isolate haustoria from infected leaves by Hahn and Mendgen (1992) made it possible to study these hallmark structures of obligate biotrophic pathogens. Building on the study of PIGs (in planta induced genes, genes found highly expressed in haustoria) (Hahn and Mendgen, 1997), more molecular and biochemical studies followed. These studies provided proof for the importance of haustoria in nutrient uptake (Voegele et al., 2001), as well as the generation of energy (Sohn et al., 2000). Among the PIGs also the first non-avirulence protein shown to be transferred from the fungus into the host cytoplasm (*Uf-*RTP1p) was discovered (Kemen et al., 2005). Current research on *U. fabae* is focused on searching novel candidate effectors among secreted proteins (Link and Voegele, 2008), augmenting the knowledge on carbohydrate uptake and metabolism, and the quest for a generally applicable method to stably transform rust fungi (Djulic et al., 2011). Keeping up this tradition of research on this species, *U. fabae* was the logical choice for generating a reference genome for the genus *Uromyces*. However, *U. fabae* could serve as a model not only for *Uromyces* species but also for other legume rusts, the most important of which at the moment is *P. pachyrhizi*.

Here, we report first results from a genome survey on *U. fabae*. Based on the results of this survey we expect to get a better understanding of the physiology of *U. fabae*. We found more candidate effectors, and we did and will do more comparisons of gene content against *Pgt* and *Pst* and also *Melampsora larici-populina* (*Mlp*). We plan to expand this survey into a full genome sequencing project.

## Sequencing

DNA was prepared from urediospores of *U. fabae* isolate I2. This isolate has been in use in the Mendgen lab (Universität Konstanz, Germany) and the Voegele lab for many years. Virtually all experiments published on *U. fabae* were made using this strain. DNA was isolated from germinated urediospores using a protocol modified from Kolmer et al. (1995). Urediospores were washed for 30 min and germinated for 3.5 h. Germinated spores were homogenized by grinding in liquid N_2_ and acid washed sand, and incubated in CTAB solution. Phenol-chloroform extraction, chloroform extraction, precipitation with 2-propanol, and RNaseA digest were performed to purify the DNA. Quality assessments showed only minor degradation and a slight bacterial contamination.

Paired end sequencing using Illumina HiSeq2000 with a 500 nt library was performed by BGI TECH SOLUTIONS (HONGKONG) CO. LIMITED (16 Dai Fu Street, Tai Po Industrial Estate, Tai Po, N.T., Hong Kong) who also supplied the assembly that is presented. 593,062,170 reads with a length of 90 bp were produced giving 53,375 Mb in raw data. 8.8% of the data were removed during filtering, leaving 48,661 Mb of clean data. The sequence reads were deposited in NCBI SRA in experiment SRX547322 corresponding to BioProject PRJNA248166.

Using SOAPdenovo reads were assembled into 59,735 scaffolds with a total length of 215,710,123 bp. N50 for the scaffolds is 5873 bp, the longest scaffold spans 72,118 bp, the shortest one 1,000 bp. These scaffolds were built from 95,847 contigs with a total length of 209,504,160 bp. N50 for the contigs is 4,171 bp, the longest contig is 45,252 bp, the shortest 200 bp. This Whole Genome Shotgun project has been deposited at DDBJ/EMBL/GenBank under the accession JNCO00000000. The version described in this paper is version JNCO01000000.

The original assembly with 1,191,649 scaffolds that was used for prediction of proteins, is very fragmented, so it is not possible to draw definite conclusions. We hope to improve our data by further sequencing (i.e., pacbio sequencing) and performing new assemblies for example with realizing mate pair sequence. One objective of this publication is to spark interest in this genome so that other groups or organizations could add their expertise into the project. However, as mentioned above, we were most interested in the gene complement, especially in distinct metabolic pathways and secreted proteins as effector candidates, so nevertheless, we set out to do further analyses that are presented below. Here we want to supply the reader with this caveat: All analyses are based on a provisional assembly.

## Genome size

Our data so far gave us three estimates on the size of the *U. fabae* genome. On the one hand we have two different k-mer analyses, a 15mer analysis that estimates the genome size to 329 Mb and a 17mer analysis that calculates to 330 Mb. On the other hand, there is the original assembly size of 422 Mb and the filtered assembly with 216 Mb. Apart from this, the genome size of *U. fabae* isolate I2 was recently measured using flow cytometry on isolated nuclei from germinated urediospores, giving an estimate of 379 Mb (Tavares et al., 2014). Given the preliminary nature of the assemblies we consider the k-mer analysis and the flow cytometric data as most reliable and thus estimate the actual genome size in the range between 330 and 379 Mb.

Analysis on other rust fungi has shown that compared to other fungi the genomes of rusts are fairly inflated. *U. fabae* seems to be no exception. The pioneering genome sequences of *Mlp* and *Pgt* also revealed a reason or the mechanism for these big genome sizes—a high amount of transposable elements (TE) (Duplessis et al., 2011). We assume that *U. fabae* likewise has a large amount of TE, probably more than *Mlp* and *Pgt*. The high amount of predicted proteins that were annotated as transposon related (see below) also seems to point in this direction. So far, an analysis of repeats and TE could not be performed, but given the large genome size, this will be an important part in the analysis of later assemblies.

## A preliminary view on the gene complement

All data regarding predicted proteins (annotation, prediction as secreted, clustering) as described below are integrated in Supplementary Table 1.

## Protein prediction and annotation

To have an idea, how useful the original assembly could be despite its fragmented state, we analyzed it with CEGMA (core eukaryotic genes mapping approach, Parra et al. (2009). This analysis showed that of the 248 highly conserved CEGs 95% were at least partially, and 89% completely present. Thus, this indicates that a large portion of the gene complement should be represented in the current assembly. Without preceding prediction and masking of repeats we used the Augustus Web server (Hoff and Stanke, 2013) with the gene structure file from the CEGMA output as a training set and 590 available ESTs (Jakupovic et al., 2006; Link and Voegele, 2008) as “hints” to predict 70,913 proteins. Compared to the 17,773 protein coding genes predicted for *Pgt*, and the 16,399 for *Mlp* (Duplessis et al., 2011), this is a gross over-prediction—most likely due in large part to TEs. For a more accurate gene prediction a better assembly, prediction of repeats, and more information on gene structure and especially more cDNA sequence information will be necessary.

To get a workable dataset steps were taken to reduce the set of predicted proteins closer to realistic numbers. First, all predicted sequences were truncated to the first methionine and all sequences shorter than 80 aa were removed. To remove redundancy among the remaining 56,594 predicted proteins they were clustered using the cd-hit-suite web server. Clustering with 0.7% ID as cutoff yielded 23,153 clusters, which seemed adequate. The representative proteins from this clustering were used for subsequent analyses.

Proteins were annotated using the Blast2GO suite. BLAST results could be obtained for 20,153 proteins, Gene Ontology (GO) terms were mapped for 14,085 proteins, and after integrating the InterProScan results and running Annex, 10,209 proteins could be annotated according to the Blast2GO rule.

The most important species among the BLAST hits in the NCBI nr database is *Puccinia graminis*, both for all hits and for best hits. The rest of the list is dominated by other fungal species, though surprisingly also plant and animal species are represented. Despite the result of the PCR on rDNA that predicted a bacterial contamination (and despite the omission of steps to remove this contamination), no bacterial species was prominent among the best hits. Almost half of the annotated proteins are transposon related indicating again that a new assembly will be necessary that should be masked against repeats with RepeatMasker.

## Families of secreted proteins/candidate effectors

Using SignalP4 760 signal peptides were predicted. Of the proteins carrying a signal peptide 135 had additional transmembrane domains (predicted by TMHMM), two were predicted as mitochondrial by TargetP, and 33 carry a predicted glycophosphatidylinositol (GPI) anchor (predGPI); six proteins have both transmembrane domains and a GPI anchor. 599 predicted secreted proteins remained.

To identify candidate effectors, which we assume to be specific to rust fungi or even to a single species, all proteins were blasted (blast+) against the protein complement of 10 basidiomycete species, among them four rust fungi, a hemibiotrophic smut fungus, a biotrophic mutualistic symbiont, a close relative of rust and smut fungi and three saprotrophic fungi (see Supplementary Tables 1, 3). Using spectral clustering (SCPS), 1,315 clusters containing at least two proteins were formed. 18,908 proteins fell into these clusters. This way, several families of secreted proteins, and also specific to rust fungi or lineage specific could be identified. For a better overview a smaller clustering just for proteins with predicted signal peptide was performed, including secretome results for *U. fabae* and predicted secreted proteins of *U. appendiculatus* and *P. pachyrhizi* (Link and Voegele, 2008; Link et al., 2014). Of the clusters that were formed 191 contained predicted secreted proteins from *U. fabae*. 44 of these families contained proteins that were found secreted with the signal sequence trap (Link and Voegele, 2008), 121 also contained *U. appendiculatus* proteins, 66 *P. pachyrhizi* proteins. Table 1 shows an overview of the 10 biggest families. Remarkably, only one of these families could be assigned a function, cluster 9, which is a family of expansins.

**Table 1:** **The 10 largest clusters of predicted secreted proteins**.

**No**.	***U. fabae***	***U. appendiculatus***	***P. pachyrhizi***	**YSST**
1	34	12	0	1
2	3	15	14	1
3	4	4	19	0
4	5	13	5	3
5	15	4	3	2
6	24	0	0	0
7	1	22	0	1
8	6	14	0	2
9	9	7	3	1
10	7	7	2	3

*Numbers indicate how many proteins of each species or how many secreted proteins determined by the yeast signal sequence trap (YSST) are present in the cluster*.

One effector family that has held our interest for some time now is the RTP (rust transferred protein) family. Our latest findings on RTP1p indicate that the protein has proteinase inhibitor function (Pretsch et al., 2013). Other findings show, that the protein forms fibrils in the extrahaustorial matrix as well as in the host cytoplasm (Kemen et al., 2013). This may be associated with slowing down cyclosis in the host cell and/or keeping the plant cell nucleus in the immediate vicinity of the haustorium, thus ensuring close contact and a better influence of the pathogen on its host. While these two functions are not mutually exclusive, as the fibrils around the haustorium could have a protective function, it is highly unusual that a protein should have both a structural and an enzymatic function. So far, structural and functional analyses have been limited to *Uf*-RTP1p.

That *Uf*-RTP1p is a member of an extended gene family was shown in recent sequencing projects. Search for *RTP1* homologs using degenerate primers yielded additional information. The most recent summary of these homologs can be found in Pretsch et al. (2013). Using tblastn to search *RTP1* homologs against our assembly we could find—in addition to *Uf-RTP1*—two more homologs. One, located on scaffold6572, shows highest similarity to *Ua-RTP2* (*Ua*: *Uromyces appendiculatus*). According to the nomenclature proposed by Puthoff et al. (2008), it will be designated *Uf-RTP2*. The second homolog showed low similarity to several *RTP1* homologs and could not be clearly assigned a best match. Using as query *RTP* homologs from other rust fungi, we also found seven homologs to *Ua-RTP9*, the highest scoring of these is located on scaffold2295 and was designated *Uf-RTP9*. The similarity between the *Uf-RTP9* homologs and *Uf-RTP1* was so low however, that they did not cluster. Therefore, we find it reasonable to designate a new family, the *RTP9* family.

It will now be interesting to do corresponding experiments with the newly identified RTP homologs to check whether these have a similar duality of functions as *Uf*-RTP1p. This phenomenon seems all the more fascinating now that *RTP* was shown to be a gene family in *U. fabae* as well as in other rust species. It seems reasonable to presume that the different proteins could be expressed at different stages and secreted from different structures, as was shown for the *Mlp-RTP*s by Hacquard et al. (2012).

In additional analyses we want to systemize the families of predicted secreted proteins into those that have functional annotation, and those that are also lineage specific, which makes them likely effector candidates. We will search the secreted proteins and the protein families for common motifs, both novel motifs and motifs already linked to effector function and/or transfer into the host cytoplasm. We will also build phylogenies for both all secreted proteins and selected families of high interest. We will also build groups of orthologs and identify those genes that have formed a high number of paralogs. Alignments of protein families will also help us to sort out proteins that were predicted as secreted because of truncations. These predictions will lead to wet-lab experiments, i.e., phenotypical screens like cell death suppression assays, search for interaction partners, silencing experiments and localizations. As indicated earlier, we will try to improve the assemblies and gene prediction and, given the opportunity, expand this survey into a full genome sequencing project.

### Conflict of interest statement

The authors declare that the research was conducted in the absence of any commercial or financial relationships that could be construed as a potential conflict of interest.
